# Low ambient humidity impairs barrier function and innate resistance against influenza infection

**DOI:** 10.1073/pnas.1902840116

**Published:** 2019-05-13

**Authors:** Eriko Kudo, Eric Song, Laura J. Yockey, Tasfia Rakib, Patrick W. Wong, Robert J. Homer, Akiko Iwasaki

**Affiliations:** ^a^Department of Immunobiology, Yale University School of Medicine, New Haven, CT 06520;; ^b^Department of Pathology, Yale University School of Medicine, New Haven, CT 06520;; ^c^Department of Pathology and Laboratory Medicine, Veterans Affairs Connecticut Healthcare System, West Haven, CT 06516;; ^d^Department of Molecular, Cellular and Developmental Biology, Yale University, New Haven, CT 06511;; ^e^Howard Hughes Medical Institute, Chevy Chase, MD 20815

**Keywords:** flu season, interferon, mucosal immunity, respiratory tract, disease tolerance

## Abstract

In the temperate regions, seasonal influenza virus outbreaks correlate closely with decreases in humidity. While low ambient humidity is known to enhance viral transmission, its impact on host response to influenza virus infection and disease outcome remains unclear. Here, we showed that housing Mx1 congenic mice in low relative humidity makes mice more susceptible to severe disease following respiratory challenge with influenza A virus. We find that inhalation of dry air impairs mucociliary clearance, innate antiviral defense, and tissue repair. Moreover, disease exacerbated by low relative humidity was ameliorated in caspase-1/11–deficient Mx1 mice, independent of viral burden. Single-cell RNA sequencing revealed that induction of IFN-stimulated genes in response to viral infection was diminished in multiple cell types in the lung of mice housed in low humidity condition. These results indicate that exposure to dry air impairs host defense against influenza infection, reduces tissue repair, and inflicts caspase-dependent disease pathology.

## Significance

Influenza virus causes seasonal outbreaks in temperate regions, with an increase in disease and mortality in the winter months. Dry air combined with cold temperature is known to enable viral transmission. In this study, we asked whether humidity impacts the host response to influenza virus infections. Exposure of mice to low humidity conditions rendered them more susceptible to influenza disease. Mice housed in dry air had impaired mucociliary clearance, innate antiviral defense, and tissue repair function. Moreover, mice exposed to dry air were more susceptible to disease mediated by inflammasome caspases. Our study provides mechanistic insights for the seasonality of the influenza virus epidemics, whereby inhalation of dry air compromises the host’s ability to restrict influenza virus infection.

Influenza A viruses (IAVs) cause seasonal infections worldwide, leading to half a million deaths annually (1, 2). IAV outbreaks occur during the winter months in temperate regions, peaking between November and March in the Northern Hemisphere and between May and September in the Southern Hemisphere (3–5). Several factors are thought to contribute to this seasonality, including fluctuations in temperature, humidity, indoor crowding, and sunlight or vitamin D exposure (5–8). A key epidemiological study analyzing data collected over 30 y across the continental United States showed that a drop in absolute humidity, which is dependent on relative humidity and temperature, correlates most closely with the rise in influenza-related deaths (9). Experimental studies in guinea pigs demonstrate that low temperature and low humidity enable aerosol transmission of influenza virus, providing one explanation for the seasonality of viral transmission (10). While these studies clearly show that environmental conditions affect transmission of influenza virus, the impact of ambient humidity on host response to influenza virus infection and disease outcome remains unclear.

During influenza infection, the respiratory mucosal barrier provides the first line of defense. The mucus layer, the surface liquid layer, and the cilia of the surface of the bronchus epithelia promote mucociliary clearance (MCC) of invading pathogens and particles. If the virus breaches these layers, the innate immune defense mechanisms triggered through recognition of viral pathogen-associated molecular patterns (PAMPs) by RIG-I and TLR7 will induce secretion of type I interferons (IFNs) to turn on hundreds of IFN-stimulated genes (ISGs) to block virus spread (11). If the virus manages to breach the innate defense layer, the adaptive immune system is engaged to induce virus-specific T and B cell immune responses critical for the clearance of IAV (12). A recent study highlights the importance of using Mx1 congenic mice to study host response to IAV infection. Most laboratory mouse strains are highly susceptible to IAV infection due to a defective *Mx1* gene, an important ISG against IAV (13, 14). Mx1 congenic mice reveal the necessity of RIG-I signaling through MAVS and TLR7 for inducing type I IFN response and controlling viral replication (15). In the absence of these sensors, compensatory activation of caspase-1/11 induces lung pathology and mortality, due to the formation of neutrophil extracellular traps (15). Thus, the Mx1 congenic mice provide a physiologically relevant model to study IAV infection and disease.

Here, we examined the impacts of relative humidity (RH) on host response to IAV infection and disease outcomes in Mx1 congenic mice. We show that mice kept at low relative humidity (10–20% RH) experience more severe symptoms than those kept at higher relative humidity (50% RH). Lower RH impaired mucociliary clearance and tissue repair and blocked the induction of ISGs known to restrict IAV, resulting in higher viral burden. Further, disease exacerbation at low RH was facilitated by inflammasome caspase activity. Thus, our data suggest that controlling the relative humidity is important to prevent influenza infection and disease outcomes in the dry winter season.

## Results

### Low Ambient Humidity Leads to More Severe Disease in Mx1 Congenic Mice.

**Fig. 1.**
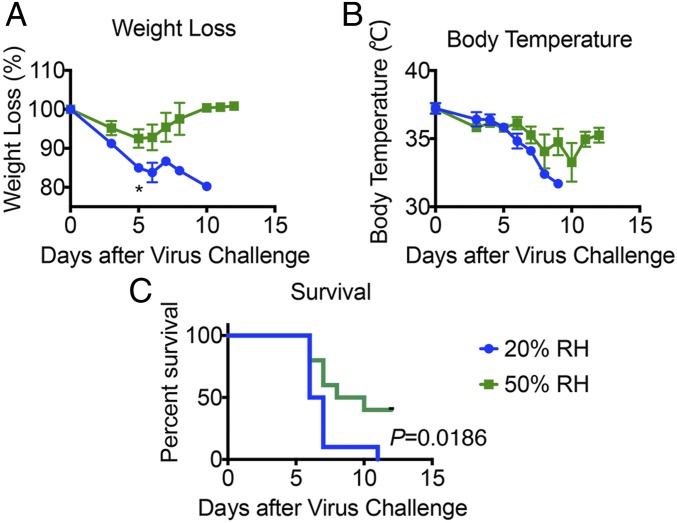
Low relative humidity predisposes Mx1 congenic mice to influenza disease. Mx1 congenic mice were preconditioned at 20% and 50% RH for 5 d and then challenged with aerosolized hvPR8 at 2 × 10^5^ pfu/mL. (*A*) Weight loss, (*B*) core body temperature, and (*C*) survival were monitored for 11 d (*n* = 10 mice per group, pooled from two independent experiments). Data are representative of five experiments and means ± SEM *P < 0.05; one-way ANOVA; log-rank (Mantel–Cox).

Mx1 mice at 50% RH were infected with varying doses of highly virulent IAV PR8 strain (hvPR8) to determine the LD_50_ (*SI Appendix*, Fig. S1). Based on this dose–response, we decided to use 2 × 10^5^ pfu for aerosol challenge to approximate LD_50_. To investigate the impact of ambient humidity on the host response to influenza infection, we employed environment chambers to precondition mice at different RH for 4–5 d at 20% or 50% RH while holding temperature constant at 20 °C before respiratory challenge with hvPR8. Immediately following infection, mice were returned to the environmental chambers and maintained in the respective RH for up to 7 d after infection. When challenged with 2 × 10^5^ pfu/mL of aerosolized hvPR8, Mx1 mice housed at 20% RH suffered a worse disease course compared with those kept at 50% RH, with more rapid weight loss, drop in body temperature, and shortened survival (Fig. 1). These data indicated that low humidity renders Mx1 mice more susceptible to IAV disease.

### Low Ambient Humidity Predisposes Mice to Caspase-1/11-Dependent Influenza Disease.

**Fig. 2.**
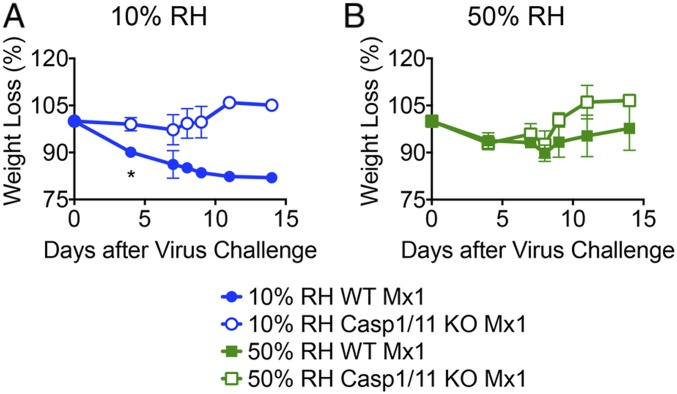
Low humidity increases influenza disease through caspase-1/11 activation. WT Mx1 mice or caspase-1/11 KO Mx1 mice were preconditioned at 10% and 50% RH for 5 d and challenged with aerosolized hvPR8 at 2 × 10^5^ pfu/mL for 15 min (*n* = 6 mice per group). (*A* and *B*) Weight loss was monitored for 14 d. Data are representative of four experiments and means ± SEM *P < 0.05; one-way ANOVA; Student’s *t* test.

Our previous study showed that caspase-1/11–dependent inflammation underlies disease pathogenesis of influenza virus in Mx1 congenic mice (15). Thus, we examined the role of these inflammasome caspases on IAV disease in mice exposed to different humidity levels. Notably, unlike WT Mx1 mice, caspase-1/11 KO Mx1 mice were spared from influenza disease exacerbation even when preconditioned in low ambient humidity of 10% RH (Fig. 2). These results indicated that dry air exposure predisposes mice to severe IAV disease in a caspase-1/11–dependent manner.

### Low Humidity Exposure Impairs Viral Clearance Independent of T Cell Immune Responses.

**Fig. 3.**
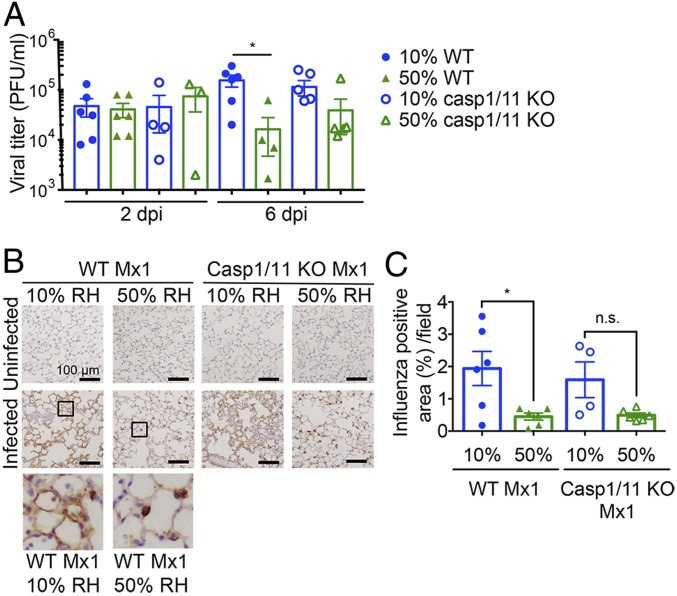
Low humidity impairs viral clearance independent of the adaptive immune response. Mx1 congenic WT mice or caspase-1/11 KO Mx1 mice were preconditioned at 10% and 50% RH for 5 d and challenged with aerosolized hvPR8 at 2 × 10^5^ pfu/mL for 15 min (*n* = 4–6 mice per group). (*A*) Bronchoalveolar lavage collected at 2 and 6 d.p.i. Data are representative of four experiments and means ± SEM. There are not significant differences except between 10% WT and 50% WT. (*B* and *C*) Mice were killed on day 6 and lung sections from each group were subjected to immunohistochemistry with an antiinfluenza A antibody (*B*). Percentage of influenza positive area was assessed by image analysis (*C*). Data are means ± SEM *P < 0.05; one-way ANOVA. n.s., not significant.

To determine whether ambient humidity alters respiratory viral clearance, we measured viral titers in the lungs of mice exposed to 10% vs. 50% RH and infected with influenza virus. While similar infectious virus doses are found in the lung at 2 d postinfection (d.p.i.), mice housed in 10% RH sustained slightly higher viral titers at 6 d.p.i. (indicative of continued viral growth), while those kept at 50% RH had reduced titers at 6 d.p.i. (indicative of viral control) (Fig. 3*A*), suggesting that higher humidity increased resistance to influenza virus. Interestingly, similar viral titers were observed in WT and caspase-1/11 KO Mx1 mice in 10% RH (Fig. 3*A*). However, caspase-1/11 KO Mx1 mice showed no significant weight loss (Fig. 2) despite the impaired resistance at 10% RH. When weight loss was plotted against viral titers, WT, but not caspase-1//11 KO Mx1 mice, showed positive correlation (*SI Appendix*, Fig. S2). These data indicated that low humidity renders mice less able to control IAV infection and that disease mediated at low humidity requires active inflammasome caspases.

To examine the cell types infected by IAV, we harvested the lungs at 6 d.p.i. and stained for viral antigen. Influenza protein was detected in both the alveolar epithelial cells and in alveolar macrophages throughout the lungs of mice exposed to 10% RH, in both WT and caspase-1/11 KO Mx1 mice (Fig. 3 *B* and *C*). In contrast, virus staining was mostly confined to the alveolar macrophages of mice housed in 50% RH, with little staining observed in the epithelial cells in both genotypes (Fig. 3 *B* and *C*).

Given the enhanced viral infection and delayed clearance at later time points in low humidity, we next examined whether 50% RH promotes a more robust adaptive immune response to confer protection against influenza infection. Since we observed differences in disease as early as 5 d.p.i. (Fig. 1), before the onset of a protective antibody response, we focused on T cell immunity to IAV. Analysis of IAV PA- or NP-specific tetramer^+^ CD8 T cells in the mediastinal lung draining lymph nodes (*SI Appendix*, Fig. S3 *A* and *B*) showed less IAV-specific CD8 T cells in mice exposed to 50% RH than 10% RH. This is presumably due to more IAV antigens being generated from higher viral burden at the 10% RH (Fig. 3). In contrast, we detected similar numbers of IAV-specific CD8 T cells in the lungs of mice infected at different humidity levels (*SI Appendix*, Fig. S3 *C* and *D*). These results collectively indicate that the protection provided by 50% RH is not through enhanced induction of T cell immune responses but more likely due to increased clearance of infectious virus by innate immune mechanisms.

### Low Humidity Exposure Impairs Tissue Repair of the Airway Epithelia.

**Fig. 4.**
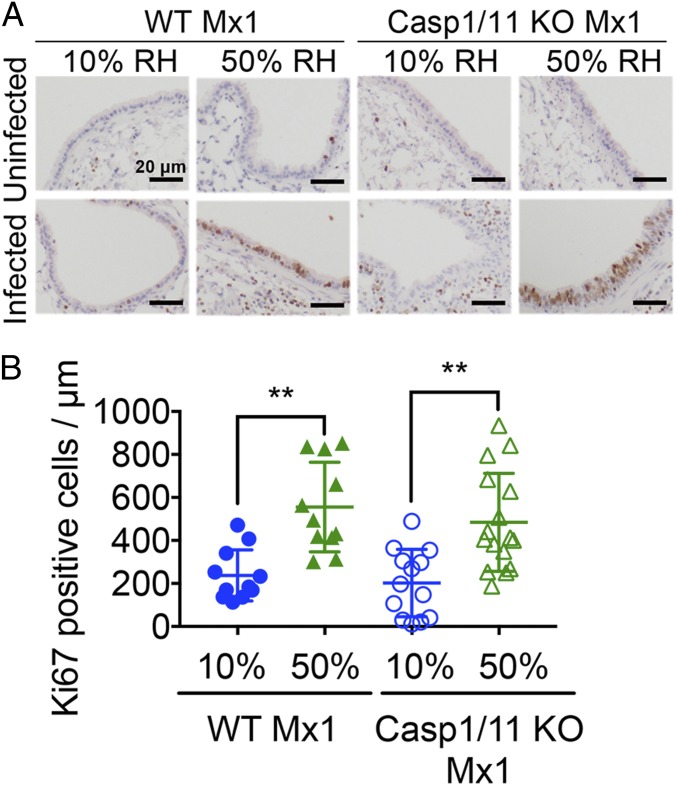
Low humidity impairs tissue repair of airway epithelial cells. (*A* and *B*) WT Mx1 and caspase-1/11 KO Mx1 mice were preconditioned at 10% and 50% RH for 5 d and challenged with aerosolized hvPR8 at 2 × 10^5^ pfu/mL for 15 min (*n* = 4–6 mice per group). Mice were killed on day 6 and lung sections from each group were subjected to immunohistochemistry with an anti-Ki67 antibody (*A*). Ki67^+^ cells were assessed by image analysis (*B*). Data are means ± SEM **P < 0.01; one-way ANOVA.

Our data thus far indicated that exposure to low RH impairs antiviral resistance. Viral spread in the lung airway epithelia is expected to result in tissue damage (16, 17). To investigate whether tissue repair mechanisms are impacted by the humidity of the inhaled air, we examined the proliferative response of lung epithelial cells before and after influenza infection. Exposure to low or normal humidity air in uninfected mice showed very little difference in epithelial proliferation (Fig. 4). In contrast, on day 6 after IAV infection, a much higher proportion of airway epithelial cells of mice housed in 50% RH were proliferative (Ki67^+^) compared with those kept in 10% RH (Fig. 4). These results suggest that the tissue repair function of epithelial cells might be impaired at 10% RH.

### Low Humidity Exposure Decreases Mucociliary Clearance in Mouse Trachea.

**Fig. 5.**
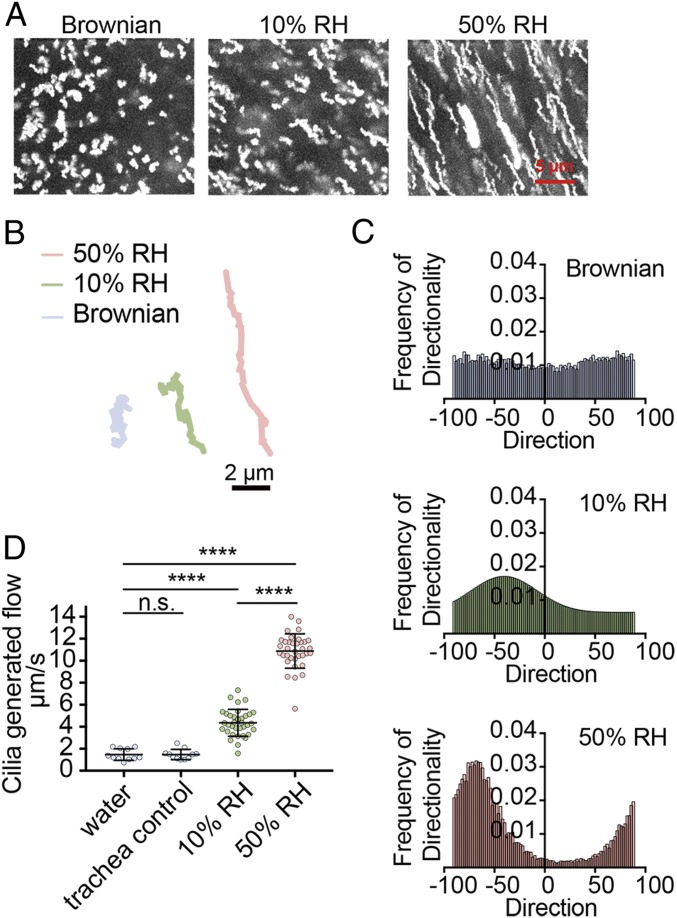
Low humidity decreases MCC. (*A*–*D*) WT Mx1 mice were preconditioned at 10% and 50% RH for 7 d, and tracheas were collected for MCC assay (*n* = 3 mice per group). Frequency of directionality and cilia-generated flow rate were measured by microscopy. (*A*) Maximum projected images of particle diffusion over a span of 1 s. (*B*) Representative particle trajectory over a span of 1 s. (*C*) Frequency chart of the directionality of particles in tracheas of Mx1 mice preconditioned at different humidity. (*D*) Cilia-generated flow was measured by multiple particle tracking. Water control was measured by diluting particles in water and loading them onto slides to simulate Brownian motion. Trachea control represents tracheas from WT mice that were collected and imaged 1 h later to ensure no flow was generated by dead tissue. The 10% and 50% RH tracheas were imaged within 5 min of being excised from mice. Data are means ± SEM ****P < 0.0001; one-way ANOVA. n.s., not significant.

Given that 10% RH exposure results in impaired viral clearance, we next examined the impact of humidity on MCC. MCC is an important innate defense mechanism which removes pathogens, allergens, and debris by ciliary action (18). To determine the impact of low humidity exposure on the function and efficiency of the mucociliary transport, we measured MCC of the trachea of mice exposed to 10% vs. 50% RH. Tracheal MCC was significantly reduced in 10% RH compared with 50% RH (Fig. 5 and Movies S1–S3). Both the directionality of flow (Fig. 5*C*) and the flow speed generated (Fig. 5*D*) were severely impaired in the trachea of mice exposed to 10% RH (Movie S1) compared with 50% RH air (Movie S2).

### Low Ambient Humidity Blocks IFN-Stimulated Gene Expression in the Lung.

**Fig. 6.**
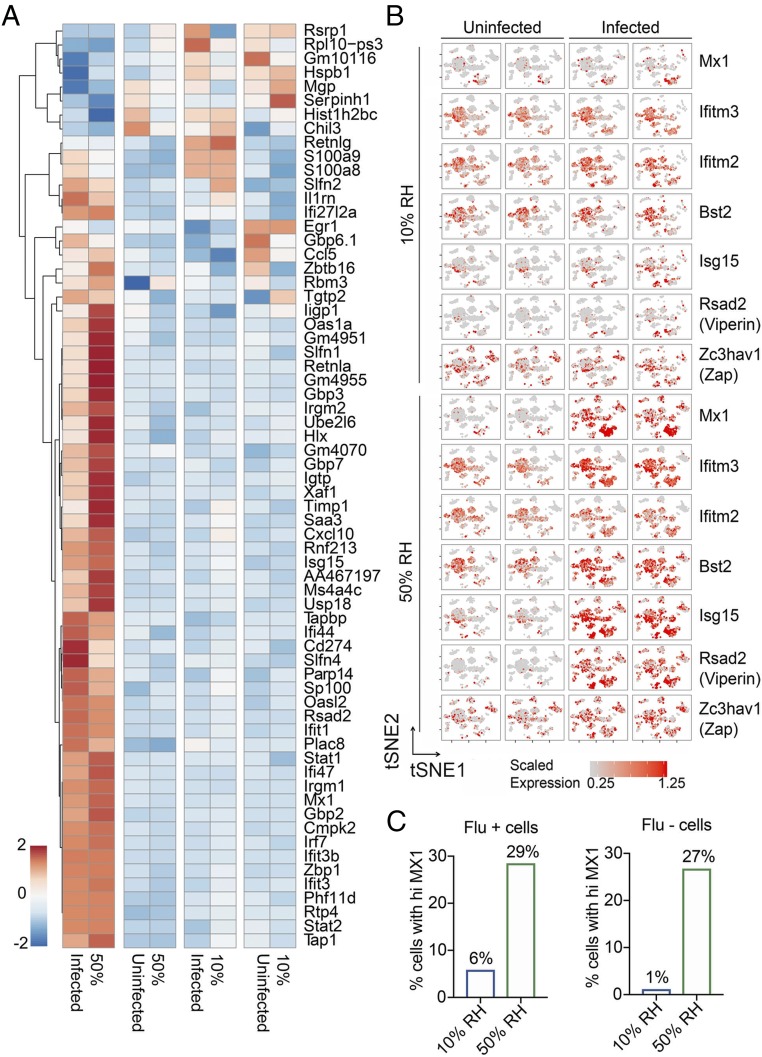
Analysis of low humidity impact by scRNA-seq. WT Mx1 mice were preconditioned at 10% and 50% RH for 7 d, and challenged intranasally with 750 pfu/mL of hvPR8. Uninfected and infected mice were killed on day 2 and lung tissue was subjected to scRNA-seq. (*A*) Differentially expressed genes in alveolar macrophages before and after infection. (*B*) Heatmap tSNE of flu-associated ISGs in different biological conditions. (*C*) Percentage of cells with Mx1 expression among the cells that are either positive or negative for influenza viral RNA.

Finally, to examine the impact of humidity on host response to IAV across multiple different cell types, we performed single cell RNA-sequencing (scRNA-seq) using samples obtained from the whole lung tissue of uninfected and IAV-infected mice housed in 10% and 50% RH. The weight of mice was monitored after infection (*SI Appendix*, Fig. S4*A*) and cell populations were analyzed by flow cytometry (*SI Appendix*, Fig. S4*B*). The scRNA-seq of whole lung single-cell suspension revealed 22 distinct cell types belonging to epithelial, endothelial, phagocytes, and lymphocyte groups based on the t-distributed stochastic neighbor embedding (tSNE) clustering (*SI Appendix*, Fig. S5). An abundance of cell types shifted in a predictable manner, such as the influx of neutrophils in infected mice (*SI Appendix*, Fig. S6*A*, blue box). In addition, we detected changes in alveolar macrophage phenotype in infected mice (*SI Appendix*, Fig. S6*A*, red box). The changes in the alveolar macrophages in infected mice were due to genes up-regulated in pathways related to defense responses [Gene Ontology (GO):0006952], response to other organisms (GO:0051707), and other expected responses against flu (Fig. 6*A* and *SI Appendix*, Fig. S6*F*). Otherwise, no large changes in the composition of immune cells, endothelial, or epithelial cell populations were detected in response to infection or exposure to different levels of humidity (*SI Appendix*, Figs. S5 and S6).

Strikingly, ISGs known to be critical in antiviral defense against IAV, namely, Mx1 (19), IFITM3 (20), IFITM2 (21), BST2 (22), Viperin (23), ISG15 (24), and ZAP (25) were all elevated in response to IAV infection across different cell types in mice exposed to 50% RH condition but not those kept in 10% RH environment (Fig. 6*B*). Of the cells that contained IAV viral RNA, a higher proportion of cells expressed Mx1 in mice housed at 50% RH compared with 10% RH (Fig. 6*C*, *Left*). Similarly, of the cells that were devoid of viral RNA, higher proportions also expressed Mx1 at 50% RH, suggesting that IFN-induced Mx1 expression is also more robust at the higher humidity (Fig. 6*C*, *Right*). Collectively, our data show that exposure of the host to low ambient humidity results in impaired MCC, reduced ISG expression and antiviral resistance, increased viral spread, tissue damage, impaired epithelial repair, and loss of disease tolerance to pathology mediated by the inflammasome caspases, leading to lethality from influenza infection.

## Discussion

Seasonality of the influenza epidemic is linked to environmental factors such as the drop in ambient humidity and temperature. Low humidity has been shown to impact the transmission of IAV from an infected host to an uninfected host (7). In this study, we examine whether relative humidity impacts host responses to influenza infection using Mx1 congenic mice. Exposure of mice to low relative humidity increased their susceptibility to more severe IAV disease and faster lethality. Low relative humidity exposure rendered mice less competent to cope with the pathological consequences of inflammasome caspase activation. Moreover, low humidity exposure impaired the MCC function of the trachea, and tissue repair function of the airway epithelial cells, resulting in viral spread and tissue damage.

One of the likely reasons by which high relative humidity prevents influenza disease is through increasing the expression levels of anti-IAV ISGs, including Mx1 (19, 26–28), IFITM3 (20, 29, 30), IFITM2 (21), BST2 (22), Viperin (23), ISG15 (24), and ZAP (25). How does inhalation of dry air result in the inability of mice to induce antiviral ISGs in response to IAV infection? One possibility is the host stress response to cope with the stress of dehydration and loss of MCC and accumulation of particles in the respiratory tract may be incompatible with IFN and ISG induction. Our recent work showed that activation of the NRF2-dependent antioxidant pathway is incompatible with the induction of antiviral IFN and restriction of respiratory virus replication (31). Future work is needed to determine the precise mechanism by which dry air exposure leads to impaired antiviral signaling.

Another reason for the inability of the host animal exposed to low humidity to clear the virus (Fig. 3*A*) may be due to the reduced MCC (Fig. 5) and removal of airway virus particles up the trachea. Our data showed that exposure to low relative humidity decreased MCC, both with respect to the direction of flow and the speed (Fig. 5). These results are consistent with the observations made in humans, that under low humidity and temperature, the length of the periciliary layer is reduced, movement of ciliary cells becomes decreased, and MCC slows down (32–34), possibly resulting in increased pathogen spread.

Mice housed in 10% RH suffered from IAV disease that was driven by caspase-1/11. Of note, caspase-1/11–deficient mice housed at 10% RH harbored similar viral burden (Fig. 3*A*), viral spread (Fig. 3 *B* and *C*), and loss of epithelial proliferation (Fig. 4) to the WT mice housed at 10% RH. However, weight loss (*i*) did not correlate with viral titers (*SI Appendix*, Fig. S2) and (*ii*) was significantly less in caspase-1/11 KO mice compared with WT mice kept at low humidity (Fig. 2), indicating that caspase-1/11 KO mice were better able to cope with the same infection and were disease tolerant. These data show that caspase-1/11–mediated pathology is downstream of viral replication and epithelial repair. Our previous study showed that in Mx1 congenic mice, caspase-1/11–driven disease pathology was only evident when the host mice were deficient in innate resistance (TLR7 and MAVS deficient) (15). Our current study showed that low humidity impaired ISG induction across different cell types of the lung, resulting in higher viral burden and caspase-1/11–dependent pathology. Taken together, disease seen in the low humidity condition may be the result of a combination of factors, including reduced ISG expression, impaired antiviral resistance enabling higher viral burden, and caspase-1/11–dependent host damage.

In summary, our study provides mechanistic insights through which ambient humidity impacts physical and innate immune defense against a respiratory viral infection. These mechanisms, such as impaired MCC and ISG induction may in part underlie the epidemiological correlation of a drop in absolute humidity preceding death from seasonal influenza infection in temperate regions (9). It is worth noting that humidity does not seem to affect host defenses against influenza viruses in all situations, as tropical and subtropical climate regions, which are wet and warm, allow for influenza virus to thrive (35, 36). Future studies will be required to understand why certain regions of the world may be affected differently by humidity and temperature. Our study suggests that increasing ambient humidity may be a viable strategy to reduce disease symptoms and to promote more rapid recovery in influenza-infected individuals.

## Materials and Methods

### Mice.

C57BL/6 mice carrying a functional *Mx1* allele (37) were used. *Caspase-1/11* KO mice crossed to Mx1 congenic mice have been previous reported (15). Mice were maintained in our facility until the ages described. All procedures used in this study complied with federal and institutional policies of the Yale Animal Care and Use Committee. The study was approved by an institutional review board.

### Environmental Conditioning and Infection of Mice.

Sex-matched 8- to 12-wk-old mice were kept in an environmental chamber (model 7000-10, Caron) for 4–5 d in which the RH was maintained at 10–20% RH for low humidity or 50% RH for normal humidity conditions. The temperature was maintained at 20 °C throughout the study. Mice were kept in the environmental chambers for up to 7 d.p.i. then moved to the ambient animal room conditions, which had a RH of 50–60% and temperature of 20–22 °C. For infection, highly virulent A/PR/8/34 (H1N1; hvPR8) IAV was delivered via intranasal or aerosol administration as indicated. The hvPR8 strain was adapted in Mx1 mice (38). Aerosol challenge was performed using an MPC aerosol nebulizer with aerosol pie cage (Braintree Scientific). Infection was performed in a biosafety cabinet at ambient animal room conditions. Before intranasal inoculation of IAV, mice were anesthetized by i.p. injection of ketamine and xylazine. Body weight, survival, and rectal temperature (MicroTherma 2T hand-held thermometer, Braintree Scientific) were monitored throughout the course of infections.

### Mucociliary Clearance Measurement.

MCC was measured according to previous publications (39, 40). Custom slides with 2-mm indentations were made to allow for whole trachea mounting. Mice were killed and tracheas were immediately removed, cut laterally to allow for the cilia to face the coverslip, and placed on the slide. Fifty microliters of FluoSpheres carboxylate, 0.2 μm crimson, 625/645 (Life Technologies) diluted 1:1,000 in PBS was placed on top of the trachea and mounted with coverslip. Within 5 min of trachea excision, the sample was imaged using an Opterra confocal microscope (Bruker) at 100 frames per second. All focus was matched to be ∼1 μm on top of the surface of the trachea, as flow rate decreases away from the surface of the tissue. Images were processed using ImageJ.

### Single-Cell RNA-Seq.

Mice were housed at either 10% or 50% RH in an environmental chamber for 5 d and infected with intranasal hvPR8 or mock infected with PBS. At 2 d.p.i., mice were killed, perfused with PBS, and lungs were isolated. The lung tissues were dissociated using 20 μg/mL DNase I, 1 mg/mL Collagenase A in RPMI at 37 °C for 30 min, followed by passage through a 70-μm filter and ACK buffer to remove any residual red blood cells. The cells were loaded onto the chromium controller (10× Genomics). Single-cell RNA-seq libraries were prepared using the Chromium Single Cell 3′ v2 Reagent Kit (10× Genomics) according to the manufacturer’s protocol. Samples were sequenced on the HiSeq4000 with 28-bp read 1, 8 bp i7 index, and 98-bp read 2. Sequencing results were demultiplexed into Fastq files using Cell Ranger (10× Genomics, 2.2.0) mkfastq function. Samples were aligned to mm10-2.2.0 10× genome with custom flu virome annotations that included all eight segments of the influenza A PR8. The count matrix was generated using the count function with default settings. An estimate of 10,976 cells were sequenced (four conditions, duplicates) with a mean read number of 266,989 and median gene number per cell of 2,069. Matrices were loaded into Seurat v2 (41) for downstream analysis. Cells with fewer than 500 unique molecular identifers (UMIs) or high mitochondrial content were discarded. Cell types were determined using previously published datasets as refs. 42 and 43. For differential expression of genes in alveolar macrophages, cells from 10% and 50% uninfected were pooled and 10% and 50% infected were pooled. This differentially expressed gene list was used to create an expression heatmap including each of the four conditions to highlight the intensity difference of each gene in the relative humidity. The dataset is available at https://www.ncbi.nlm.nih.gov/bioproject/PRJNA528197 (44).

### Statistical Analysis.

The data were analyzed by Student’s *t* test, one-way ANOVA with either Tukey’s or Kramer’s multiple comparison test, or log-rank (Mantel–Cox) tests. All statistical tests were calculated using GraphPad Prism (GraphPad software). A *P* value of <0.05 was considered statistically significant.

## Supplementary Material

Supplementary File

Supplementary File

Supplementary File

Supplementary File

Supplementary File

Supplementary File
