# Supplementary material for: Low ambient humidity impairs barrier function and innate resistance against influenza infection
Source: Proc Natl Acad Sci U S A. 2019 May 13;116(22):10905–10. doi: 10.1073/pnas.1902840116 (PMC6561219; doi:10.1073/pnas.1902840116)
Supplement: Supplementary File [file pnas.1902840116.sapp.pdf]

PNAS
www.pnas.org

Supplementary Information for

Low ambient humidity impairs barrier function and innate resistance against influenza infection

Eriko Kudo1, Eric Song, Laura J. Yockey, Tasfia Rakib, Patrick W. Wong, Robert J. Homer, Akiko Iwasaki

Corresponding author: Akiko Iwasaki
Email: akiko.iwasaki@yale.edu

This PDF file includes:

Supplementary text
Figs. S1 to S6
Dataset S1 to S2
Video S1 to S3

www.pnas.org/cgi/doi/10.1073/pnas.1902840116

Supplementary Information Text

Supplementary Materials and Methods

Antibodies

Antibodies to CD8-BUV395 (563786), CD3-BUV737 (564380) and CD4-BUV496 were purchased from BD Biosciences; CD127-AF488 (135017), CD44-AF700 (103026), PD1-PerCP-Cy (135207), CD11b-Biotin (101204), MHC II-AF700 (107622), CD45.2-PE (109807), CD64-PerCP-Cy (139307), CD11c-BUV605 (117329), XCR1-APC (148205), CD19-BUV605 (115540), Ly6G-AF594 (127636), CD24-APC-Cy7 (127636) and Tbet-BV711 (644817) from BioLegend; KLRG1-PE-Cy7 (25-5893-80), Ly6C-FITC (11-5931-82) and NK1.1-PE-Cy7 (25-5941-81) from eBioscience; Eomes-PE (12-4875-82) from InVitrogen; CXCR5-Biotin (551960) from Pharmingen; and IAV (ab20841) from abcam.

Measurement of viral titers

Bronchoalveolar lavage (BAL) was collected for measurement of virus titers by washing the trachea and lungs with 1 mL of PBS containing 0.1% BSA. The virus titer was measured as follows; aliquots of 100 µl of serial 10-fold dilutions of the BAL by PBS containing 0.1% BSA were added to the culture of Madin-Darby Canine kidney cells in 12-well plates. After 1 h of incubation, each well was overlaid with 1 mL of agar medium for 48 h. Cell monolayers were then stained with 0.1 % crystal violet in 20 % ethanol and plaques were enumerated.

Preparation of lung and lymph node single-cell suspensions

To obtain single lung cell suspensions, lungs were perfused with 10 mL PBS through the right ventricle, minced using razor blades, and incubated in HBSS containing 5 mM HEPES and 1.3 mM EDTA at

37 °C for 30 min. The cells were resuspended in RPMI containing 5% FBS, 1 mM $CaCl_2$, 1 mM $MgCl_2$, 5 mM HEPES, and 0.5 mg/ml collagenase D (Roche) and incubated at 37 °C for 60 min. To obtain single lymph node cell suspensions, draining lymph nodes were incubated in RPMI containing 10% FBS, 0.4 mg/ml collagenase D (Roche) at 37 °C for 60 min. The cells were incubated in 5 mM EDTA for 5 min. The resulting cells were filtered through a 70-μm cell strainer (BD Biosciences). A single-cell suspension was prepared after red blood cell lysis and analyzed by flow cytometry.

Flow cytometry

The single cell suspensions were stained with antibodies, or with MHC class I tetramers; influenza viral polymerase (PA) (SSLENFRAYV) (1) and nucleoprotein (NP) (ASNENMETM) (2) Live cells were gated based on Live/Dead staining (Thermo Fisher Scientific) and cells were analyzed on LSR II cytometer (BD Biosciences). Data were analyzed using the FlowJo software (Tree Star, San Jose, CA, USA).

Histology and immunohistochemistry

Lung tissue were fixed in 4% paraformaldehyde (PFA) overnight at 4 ℃, and embedded in paraffin blocks and sectioned. For immunohistochemistry, slides were stained for IAV or Ki67 at the Yale Pathology Tissue Services Core. Specific antibody staining was detected using HRP-conjugated secondary antibody and detected with the DAB. Slides were counterstained in hematoxylin, dehydrated, cleaned and mounted with resinous mounting media. Images were processed using ImageJ (MTrack J and Mosaic plug-in).

Supplementary Figures

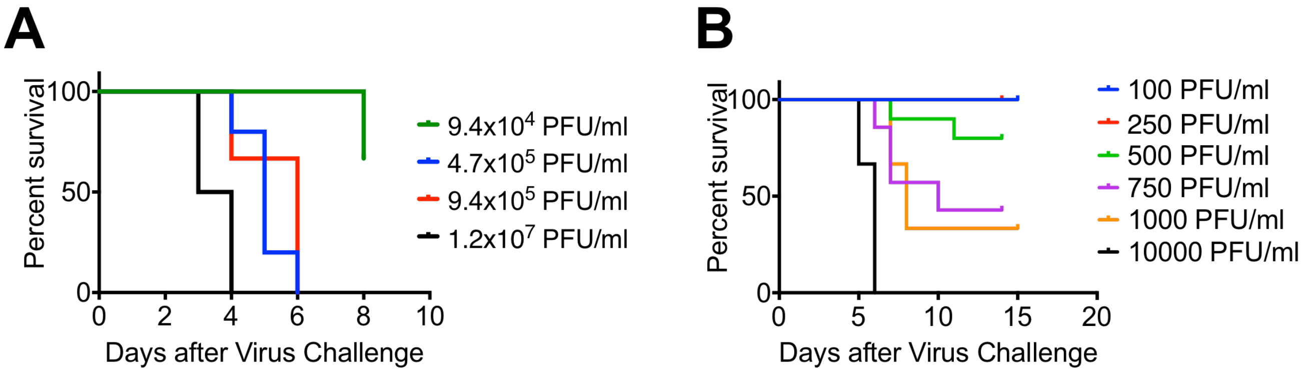

Figure S1. Dose-dependent lethality of respiratory hvPR8 infection.

Wild-type Mx1 mice kept at ambient humidity (50% RH) were challenged with aerosolized (A) or intranasal (B) with the indicated doses of hvPR8. Survival was monitored

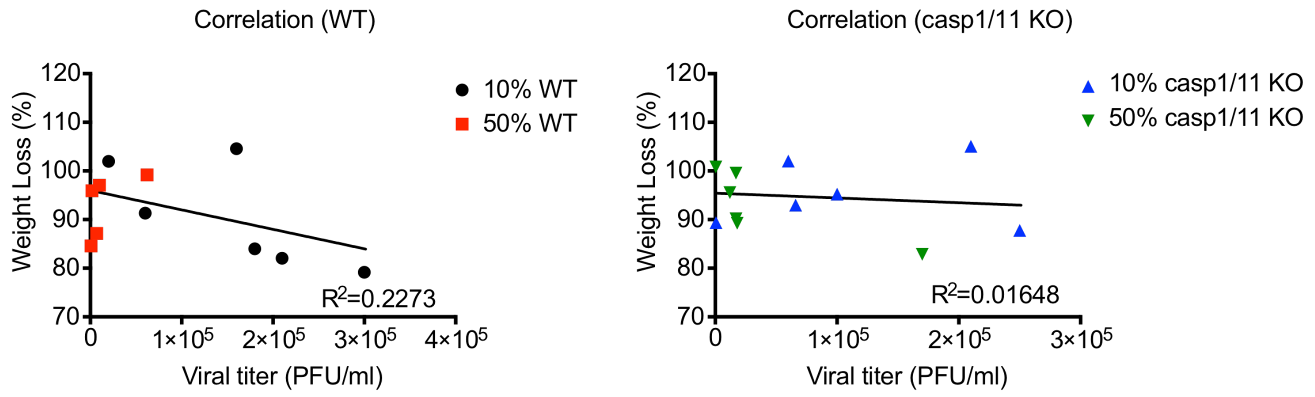

Figure S2. Correlation between weight loss and viral titer in mice infected at different humidity conditions.

Mx1 congenic wild-type mice or caspase-1/11 KO Mx1 mice were preconditioned at 10% and 50% RH for 5 days, and challenged with aerosolized hvPR8 at 2 x 10^5 PFU/ml for 15 minutes (n = 5-6 mice per group). The correlation between weight loss and viral titer at 6 dpi are plotted by and linear curve fit was analyzed using GraphPad Prism.

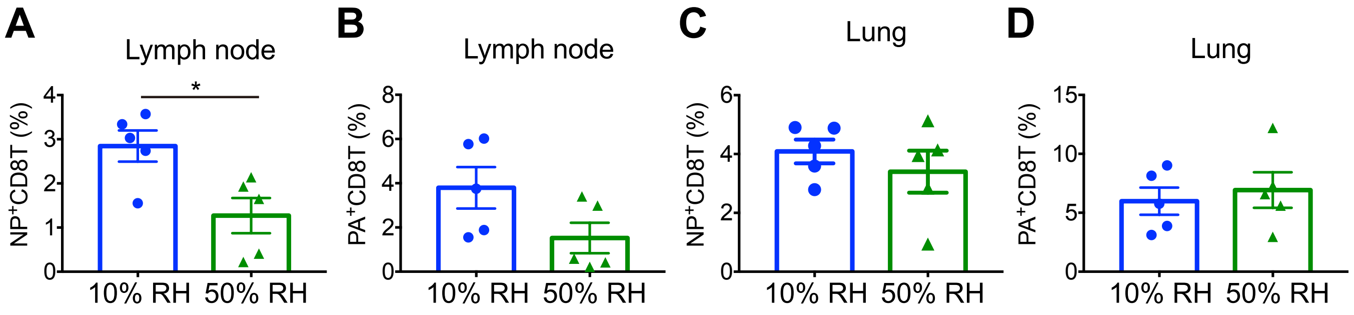

Figure S3. CD8 T cell immune response to influenza virus antigens in mice housed at different relative humidity conditions.

Mx1 congenic wild-type mice or caspase 1/11 KO Mx1 mice were preconditioned at 10% and 50% RH for 5 days, and challenged with aerosolized hvPR8 at 2x10^5PFU/ml (n=4-6 mice per group). (A-D) Influenza NP-specific or PA-specific tetramer positive CD8 T cells were measured on 8 day after infection from draining lymph nodes (A and B) or lungs (C and D). Data are means ± SEM. *P<0.05; one-way ANOVA: student's t-test.

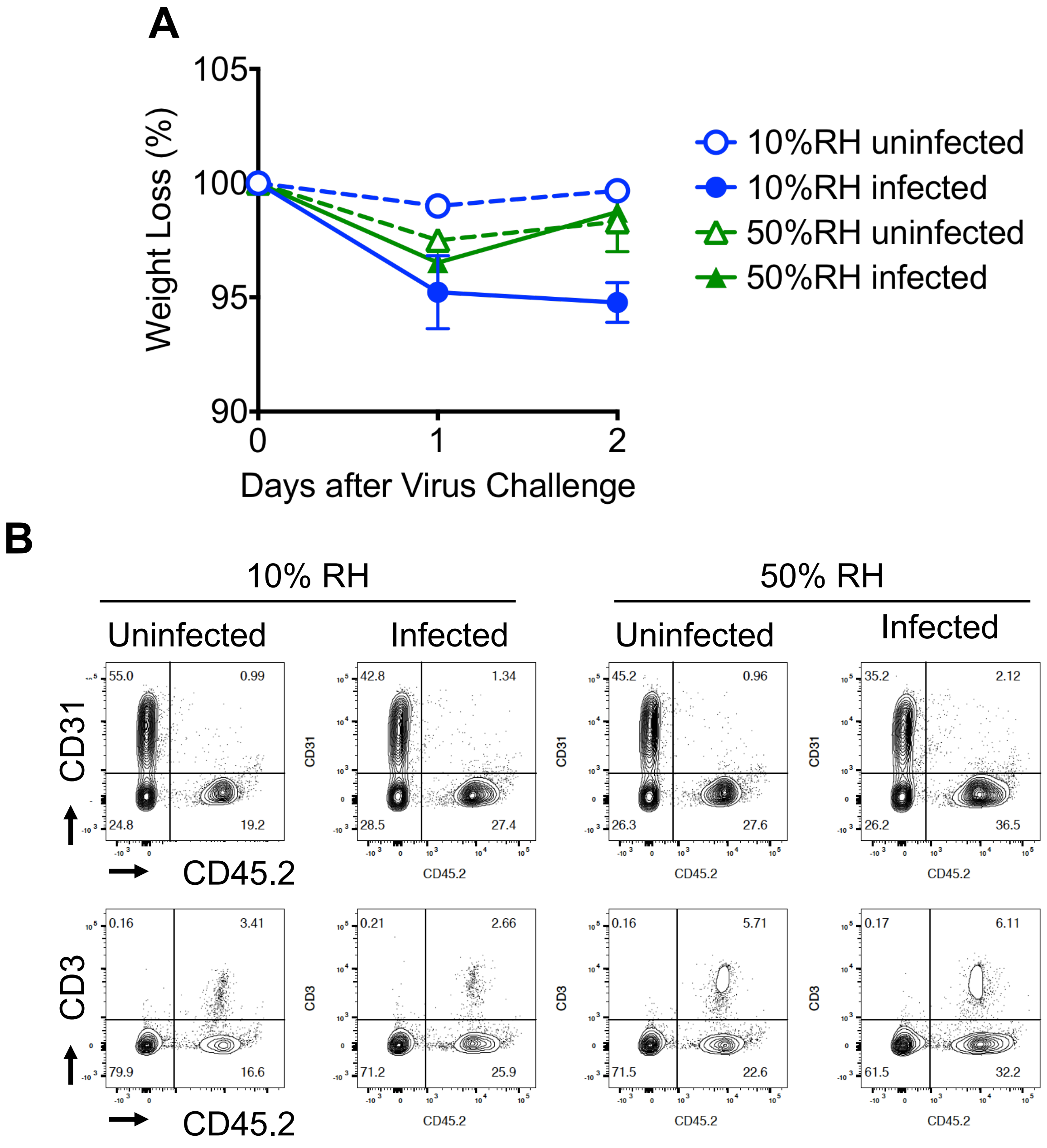

Figure S4. Phenotype of mice and cells used for ssRNAseq analysis.

Wild-type Mx1 mice were preconditioned at 10% and 50% RH for 7 days, and challenged intranasally with 750PFU/ml of hvPR8 (n=3 mice per group). **(A)** Weight loss monitor for 2 days prior to sacrificing for RNAseq analysis. Data are means ± SEM. **(B)** Lung were collected on day 2 p.i. for cell populations analysis prior to RNAseq analysis.

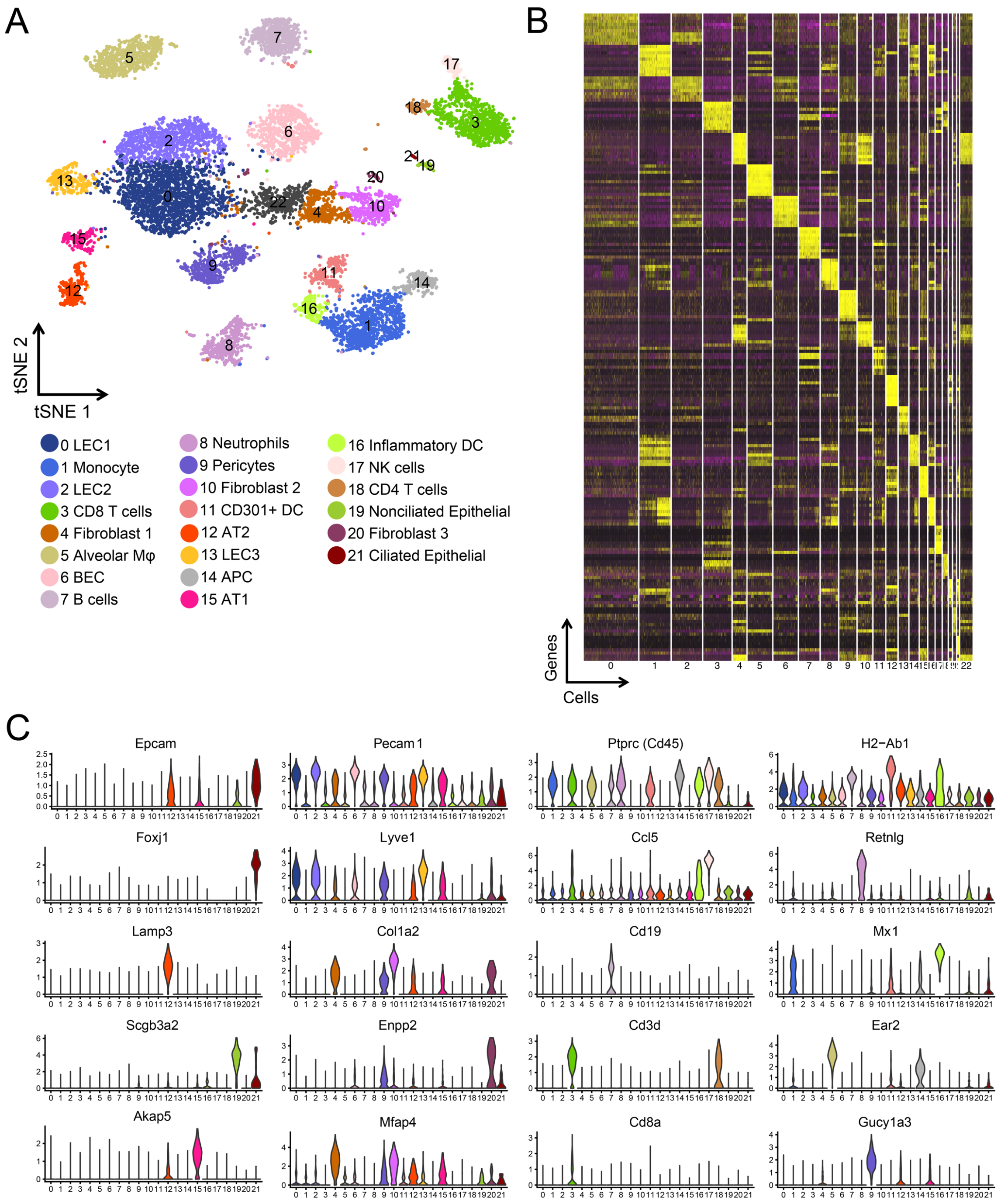

Figure S5. Single cell RNAseq analysis of cells isolated from the lungs of mice infected with influenza A virus housed in low or normal relative humidity conditions.

Wild-type Mx1 mice were preconditioned at 10% and 50% RH for 7 days, and challenged intranasally with 750PFU/ml of hvPR8. Uninfected and infected mice were sacrificed on day 2 and lung tissue subjected to single cell RNA-seq. (A) tSNE plot of four conditions tested in experiments. The red box indicates alveolar macrophages and blue box indicates neutrophils discussed in manuscript. (B) Heatmap of genes enriched in each cluster, (x-axis = cells, y-axis = genes). (C) Violin plots of representative genes used to determine cell subsets.

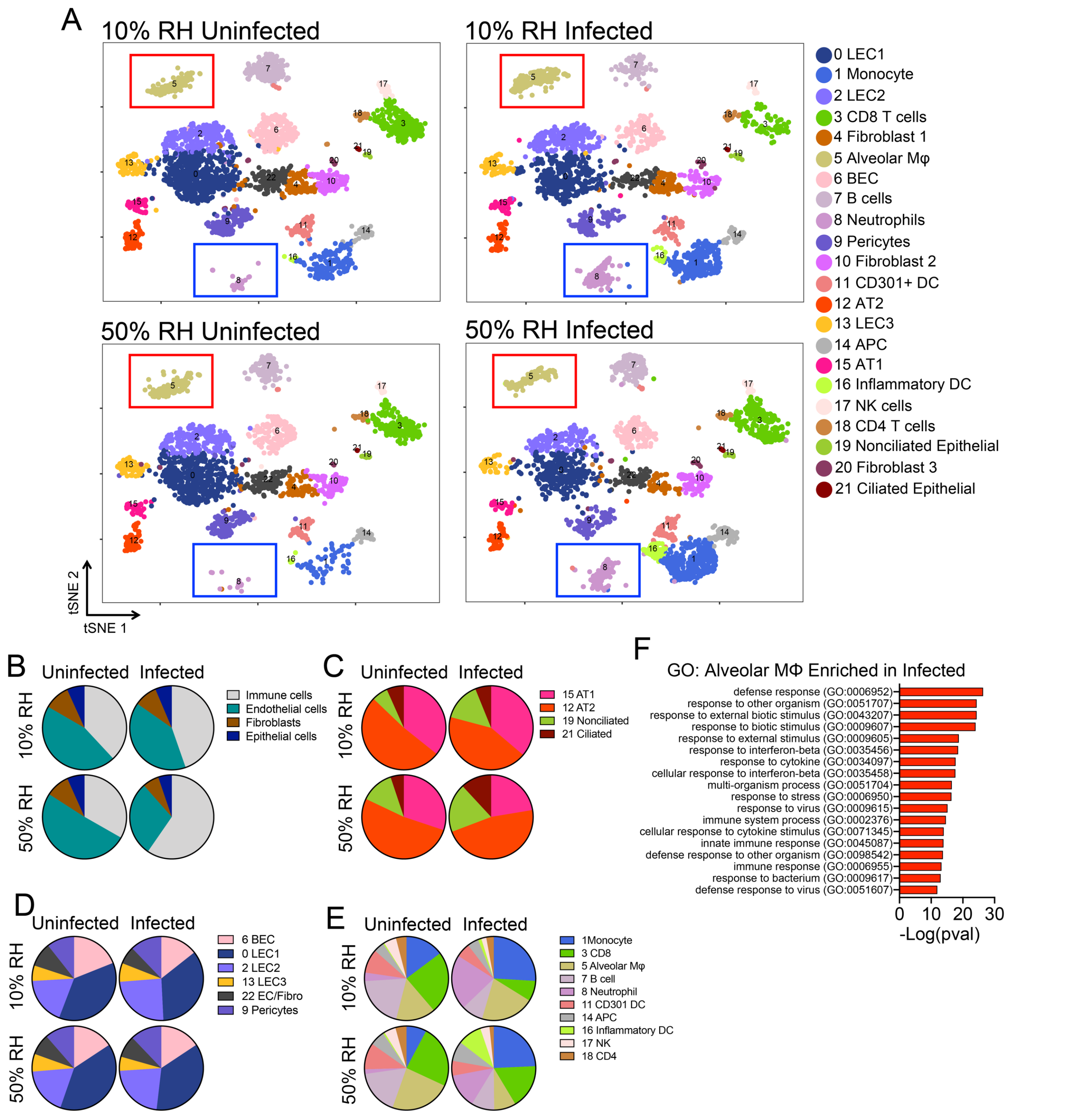

Figure S6. Analysis of lung from influenza infected mice in low and normal relative humidity conditions using single cell RNAseq.

Wild-type Mx1 mice were preconditioned at 10% and 50% RH for 7 days, and challenged intranasally with 750PFU/ml of hvPR8. Uninfected and infected mice were sacrificed on day 2 and lung tissue subjected to single cell RNA-seq. (A) tSNE plot of all cells collected in study. (B-E) Proportion of cells identified as each population. (F) GO pathway analysis of genes upregulated in (A) the infected alveolar macrophages.

Video s1

Wild-type Mx1 mice were preconditioned at 10% RH for 7 days, and tracheas were collected for MCC assay (n = 3 mice per group). Videos of images analyzed in Figure 4.

Video s2

Wild-type Mx1 mice were preconditioned at 50% RH for 7 days, and tracheas were collected for MCC assay (n = 3 mice per group). Videos of images analyzed in Figure 4.

Video s3

Tracheas of wild-type Mx1 mice were collected, and after a few hours, Brownian motion of dead tissue was analyzed by MCC assay (n = 3 mice per group). Videos of images analyzed in Figure 4.

Dataset s1 norm_combined

Normalized counts of genes from each cell population

Dataset s2 (190128 DE_EK)

Differentially expressed genes in each cluster (Figure S5B)

References

1. Pang IK, Ichinohe T, & Iwasaki A (2013) IL-1R signaling in dendritic cells replaces pattern-recognition receptors in promoting CD8(+) T cell responses to influenza A virus. *Nature immunology*.
2. Morimoto J, *et al.* (2011) Osteopontin Modulates the Generation of Memory CD8(+) T Cells during Influenza Virus Infection. *J Immunol* 187(11):5671-5683.
